# Integrating blood eosinophils and exhaled nitric oxide (FeNO) in asthma diagnostic pathways for adults and children: the PROPULSION SANTÉ observational study with translational sub-studies (DIVE, DIVE2)—protocols

**DOI:** 10.1136/bmjresp-2025-003750

**Published:** 2025-11-27

**Authors:** Morgane Gronnier, Laurence Désy, Lauranne Pouliot, Sarah-Ève Lemieux, Félix-Antoine Vézina, Philippe Lachapelle, François-Pierre Counil, Martine Duval, Dominic Cliche, Samuel Lemaire-Paquette, Lucien Coulibaly, Catherine Hudon, Larry C Lands, Francine Monique Ducharme, Sze Tse, Simon Couillard

**Affiliations:** 1 Faculté de Médecine et des Sciences de la Santé, Université de Sherbrooke, Sherbrooke, Quebec, Canada; 2 Centre Hospitalier Universitaire d’Amiens, Amiens, France; 3 Centre Hospitalier Régional de Trois-Rivières, Trois-Rivières, Quebec, Canada; 4 Faculté de Médecine de l'Université de Montréal, Montreal, Quebec, Canada; 5 Centre de Recherche du CHUS, Sherbrooke, Quebec, Canada; 6 CIUSSS de l’Estrie CHUS, Sherbrooke, Quebec, Canada; 7 Montreal Children’s Hospital-McGill University Health Centre, McGill University, Montreal, Quebec, Canada; 8 Centre Hospitalier Universitaire Sainte-Justine, Université de Montreal, Montreal, Quebec, Canada; 9 Department of Pediatrics and of Social and Preventive medicine, University of Montreal, Montreal, Quebec, Canada

**Keywords:** Asthma, Inflammation, Respiratory Function Test, Eosinophil Biology, Asthma in primary care

## Abstract

**Introduction:**

Diagnosing asthma requires confirmation by bronchodilator reversibility (BDR) with or without bronchial provocation testing (BPT). Despite being needed in 80% of suspected cases following BDR, BPT access remains limited in Canada. Type-2 (T2) inflammatory biomarkers (fractional exhaled nitric oxide (FeNO) and blood eosinophil count (BEC)) may be underutilised for BPT prioritisation and are insufficiently studied to support asthma diagnosis in real-world primary care, especially in children. We aimed to explore whether T2 biomarker-based prioritisation of BPT reduces diagnostic delays, and improves triage efficiency and guidance based on exacerbation risk.

**Methods and analysis:**

Three academic centres in Québec will measure inflammatory biomarkers alongside BDR and/or BPT interpretation for patients with suspected asthma referred by primary care providers (PROPULSION SANTÉ, NCT06981169). Consenting patients aged ≥6 years will undergo FeNO, BEC and BDR testing. If BDR is non-diagnostic, subsequent BPT will be prioritised for patients with ≥1 elevated biomarker (BEC ≥300 /µL, (FeNO ≥25 ppb if ≥12 years or FeNO ≥20 ppb if <12 years)); others will follow usual timelines. Reports to the referring healthcare providers will include standard interpretation of BDR/BPT and biomarker results to state exacerbation risk and suggested corticosteroid dosage. Asthma control and quality of life will be assessed at baseline and remotely at 4, 8 and 12 months. The primary outcome will be the delay from the reception of test request for the diagnosis of asthma patients with versus without elevated biomarker(s). Secondary outcomes include biomarkers’ diagnostic performance, asthma control, quality of life, health benefits, cost-effectiveness, environmental impact and patient satisfaction. We aim to recruit 1500 patients to PROPULSION SANTÉ, with optional biobanking for translational sub-studies for 123 adults (DIVE, NCT05992519) and 123 children (DIVE2, NCT07011394).

**Ethics and dissemination:**

Study protocols were ethically approved CIUSSS de l'Estrie–CHUS #MP-31-2025-5593/MP-31-2024-5346; 2023–4791). Results will be communicated and submitted to peer-reviewed journals.

**Trial registration number:**

ClinicalTrials.gov (NCT06981169; NCT05992519; NCT07011394).

**WHAT IS ALREADY KNOWN ON THIS TOPIC**

- Asthma diagnosis faces critical access barriers despite its high prevalence. Type-2 inflammatory biomarkers effectively predict disease risk and treatment response in asthma, but are not specific for asthma: they remain underutilised in primary care and in diagnostic settings.

**WHAT THIS STUDY ADDS**

- This study evaluates whether biomarker-based patient prioritisation can overcome diagnostic constraints by streamlining the testing pathway to earlier asthma diagnosis and enhancing interpretation reports with individualised risk estimates, potentially supporting the early identification of patients with high-risk asthma.

**HOW THIS STUDY MIGHT AFFECT RESEARCH, PRACTICE OR POLICY**

- Findings could transform the asthma diagnostic pathway and management strategies by establishing a more efficient and personalised approach that prioritises limited diagnostic resources toward high-risk patients. This may reduce delays, enhance the appropriateness of testing and support more sustainable care by limiting unnecessary procedures.

## Introduction

Asthma is among the most prevalent chronic diseases worldwide and the most common in children.^1^ In Quebec, around 10% of the population—approximately 900 000 individuals, including 300 000 children—are affected.^2 3^ Asthma is a major cause of absenteeism, healthcare expenditures and morbidity, placing a significant burden on both the healthcare system and society; yet it remains disproportionately under-researched.^4 5^

Characterised by airway inflammation and bronchial hyper-responsiveness, asthma causes nonspecific and variable symptoms, complicating diagnosis.^6^ The disease is heterogeneous, comprising several phenotypes and endotypes.^7^ In the era of precision medicine, identifying type 2 (T2) inflammation – present in half of asthmatics – is particularly important.^8 9^

T2 inflammation is mediated by T-helper type 2 (Th2) lymphocytes and group 2 innate lymphoid cells (ILC2), which secrete interleukin (IL)−4, IL-5 and IL-13, promoting eosinophil recruitment, mucus hypersecretion and is associated with epithelial damage. Non-invasive biomarkers, such as fractional exhaled nitric oxide (FeNO) and blood eosinophil count (BEC), reflect IL-4/IL-13 and IL-5 activity, respectively.^9^,^14^ Both are linked to poor prognosis and may be used to predict asthma exacerbations and accelerated lung function decline.^15 16^ In adults, the ORACLE-2 meta-analysis showed that FeNO >25 parts per billion (ppb) and BEC >300/μL are linked to increased risk of exacerbations.^15^ In children over 6 years, post-hoc analysis from the VOYAGE study found that FeNO >20 ppb and BEC >300/μL predicted higher rates of severe exacerbations.^17^,^19^

Objective testing is essential for accurate asthma diagnosis, especially given high rates of overdiagnosis estimated at 33% in adults and 44% in children in Canada.^20^ Relying on subjective clinical criteria for asthma diagnosis has led to disease overestimation, impacting treatment decisions and healthcare resources.^21^ Spirometry is recommended to demonstrate post-bronchodilator reversibility (BDR), an increase in forced expiratory volume in 1 s (FEV1) ≥200 mL and ≥12% in adults and ≥12% in children and adolescents).^6^ However, spirometry is inconclusive in up to 80% of cases (and over 60% in children), necessitating a methacholine bronchial provocation test (BPT).^22 23^ Furthermore, identifying post-bronchodilator reversibility is of doubtful clinical consequence, as associated with other diseases (eg, chronic obstructive pulmonary disease) and unrelated to the risk of asthma attacks.^24 25^

Methacholine BPT is a relatively cumbersome procedure, requiring monitoring by specialised hospital units and significant testing time, which limits accessibility. These constraints contribute to both over-diagnosis and under-diagnosis, with significant individual, societal, environmental and economic consequences.^1421 26^,^28^

Inadequate access to objective tools in primary care delays or impairs treatment, worsening asthma control, quality of life and healthcare costs.^6 21^ Zafari *et al* estimated that improved diagnostic algorithms reducing sub-optimal asthma control by 10% could save $50.5 billion over 20 years in Canada.^4^

European guidelines recently incorporated FeNO as an effective alternative and cost-effective diagnostic tool for asthma and to optimise access to specialised testing : a FeNO level ≥40 ppb is 90% specific for asthma diagnosis in adults.^29 30^ The United Kingdom’s National Institute for Health and Care Excellence (NICE) guidelines recommend using FeNO and BEC early in the diagnostic algorithm as a first-line diagnostic tool before spirometry or peak flow monitoring.^29 30^ In children ≥5 years, only FeNO (≥35 ppb) has been validated as a first-line diagnostic test, while BEC had been used after BDR and PEF, along with skin prick tests or specific IgE measurement.^29 31^ The introduction of FeNO testing as a first-line diagnostic tool is estimated to enable approximately 29 000 additional asthma diagnoses annually, leading to earlier treatment initiation, improved disease control, reduced absenteeism and potential cost savings of £100–204 million per year.^32^

In Quebec, over 3000 diagnostic tests are still pending across Centre Hospitalier Universitaire de Sherbrooke (CHUS), CHU Sainte-Justine (CHUSJ) and the Montreal Children’s Hospital (MHC), including 1300 tests for children, with wait times exceeding 1 year. BPTs are scheduled chronologically, with priority only for internal referrals, that is, for specialists of the institution. The lack of robust prospective data on FeNO and eosinophils (and limited FeNO access) has restricted their integration into diagnostic algorithms.

Our overarching aim is to address an evidence gap by evaluating the integration of inflammatory biomarkers into referral for the asthma diagnosis specifically from primary or community care under real-world conditions. Specifically, we aim to assess whether FeNO and BEC can improve the prioritisation of patients for bronchial provocation testing (BPT), thereby streamlining referrals and supporting more timely and cost-effective asthma diagnosis. Propulsion Santé also includes two translational sub-studies: DIVE (in adults) and DIVE-2 (in children) involving additional sampling (serum and nasal epithelial lining fluid (NELF)) to link inflammatory mediators (alarmins, interleukins and eicosanoids), diagnosis and T2 biomarkers to gain mechanistic insight at the point of diagnosis in primary care.

## Methods and analysis

### Study design

**Figure 1:**
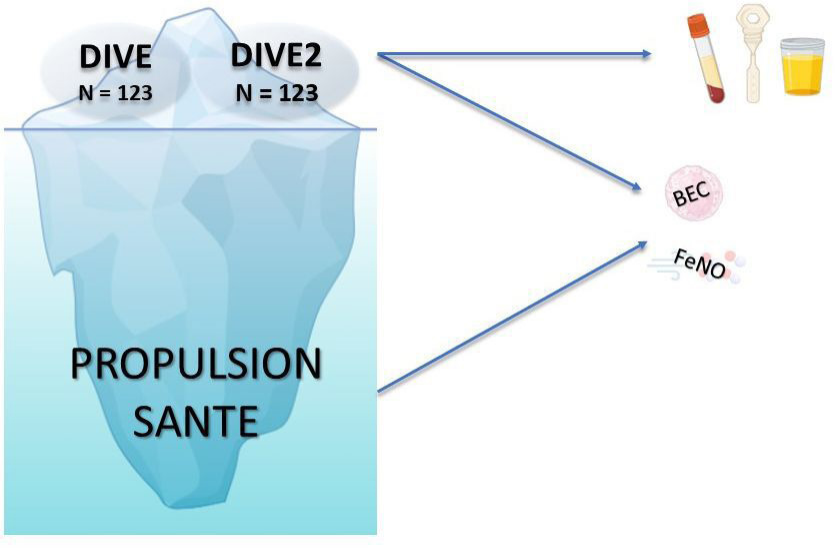
Overlap between the Propulsion Santé and DIVE studies. All patients referred by primary care for suspected asthma will be invited to participate in PROPULSION SANTÉ, forming the *base* of the iceberg, representing the broader population. The *tip* represents the DIVE (≥18 years) and DIVE-2 (6–17 years) studies, which include a smaller group of patients invited based on more selective inclusion and exclusion criteria. These two translational research studies focus on investigating type 2 inflammatory pathways through the analysis of serum, NELF and urine samples. These two translational research studies focused on investigating type 2 inflammatory pathways through the analysis of serum, NELF and urine samples. BEC, blood eosinophil count; FeNO, fraction of exhaled nitric oxide; NELF, nasal epithelial lining fluid.

PROPULSION SANTÉ (NCT06981169) is a prospective multicentre observational study conducted in patients aged ≥6 years referred by primary care for a diagnostic respiratory function test (BDR or metacholine BPT) for suspected asthma. Participants will undergo FeNO and BEC measurements during their visit for stratification. If BDR is non-diagnostic, patients will undergo a methacholine BPT and may also be invited to participate in one of the two translational sub-studies—DIVE for adults (NCT05992519) and DIVE2 for children (NCT07011394)—the latter sub-studies involve additional procedures, including blood sample collection and non-invasive nasal sampling using nasosorption (figure 1). Participants will also have the option to preserve their data and/or samples in the Quebec Respiratory Health Research Network Biobank (www.biobanque.ca).

### Setting

The study will be conducted at three Quebec medical centres: CHUS, CHUSJ and MHC-MUHC. A dedicated research respiratory therapist will be assigned to each centre for 1 year to facilitate recruitment through convenience sampling. With participant consent, longitudinal follow-up by email will occur at 4, 8 and 12 months after the initial diagnostic visits.

### Participants

Eligible participants are individuals aged ≥6 years who present with symptoms suggestive of asthma and have been referred by primary care (family physicians and nurse practitioners) or community practice (eg, paediatricians and other non-pulmonologist physicians) for asthma diagnosis at one of the three participating centres. Participants are identified from an existing waiting list of approximately 3000 patients awaiting respiratory function testing. The only exclusion criterion for PROPULSION SANTÉ is refusal to participate in the study.

Among these patients, a target sample of 123 adult participants (aged≥18 years) and 123 paediatric participants (aged 6–18 years) undergoing BPT will be consecutively invited to participate in the DIVE and DIVE-2 sub-studies, respectively, regardless of their T2 status. DIVE/DIVE2 inclusion requires non-diagnostic spirometry and no contraindication to methacholine challenge. Patients will be excluded from participation if they meet any of the following conditions: use of inhaled or systemic corticosteroids within 48 hours prior to testing; tobacco use within the previous 6 hours; diagnosis of chronic obstructive pulmonary disease (COPD), defined as all of the following — age ≥40 years, fixed airway obstruction on spirometry (FEV1/Forced Vital Capacity (FVC)<0.7) and a smoking history >10 pack-years or documented alpha-1 antitrypsin deficiency— and evidence of parenchymal lung disease (such as cystic fibrosis or bronchiectasis), unstable cardiac disease or a history of acute respiratory infection (viral or bacterial) within the preceding 4 weeks.

### Study visits

Study visits will coincide with medically recommended testing for suspected asthma (pre-/post-bronchodilator spirometry followed, if negative, with methacholine). Biobanking for DIVE and DIVE2 will only be conducted during methacholine visits. Electronic informed consent (and assent when applicable) will be obtained via REDCap.^33 34^ Additional data will be gathered on asthma exacerbation history and healthcare resource utilisation over the preceding year, including medications, by the use of reMed,^35^ a provincial research data repository of community pharmacy dispensed drugs that covers individuals under the public drug insurance plan.

During the single study visit, baseline bio-anthropometric and clinical data (age, sex, ethnicity, height and weight) will be collected. Participants will be interviewed regarding their medical history (smoking status, respiratory medication (with simultaneous carbon footprint calculation based on the number and type of inhalers delivered), recent (< 6 last weeks) respiratory infection and allergic history. Patients (and their legal guardian, if applicable) will complete standardised questionnaires on asthma control for the last 4 weeks (Asthma Control Questionnaire 5 (ACQ-5),^36^ Asthma Control Test (ACT)^37^ in adults and childhood Asthma Control Test (c-ACT) for children^38^ and of quality-adjusted life years (QALY) (EuroQol Five Dimensions Five Levels (EQ-5D-5L)^39 40^ for adults and EuroQol Five Dimensions Youth (EQ-5D-Y)^41^ for children).

Research respiratory therapists will first measure FeNO during a 10 s exhalation (6 s for children unable to cooperate, with a 10- s expiration)^31 42^ using NIOX VERO device (approved by Health Canada as a clinical tool). The latter was selected based on its established precision (coefficient of variation <10%), reproducibility and clinical validation.^43 44^ Then, blood samples will be collected for BEC either via capillary puncture (preferred for children), using the single-use Tasso+blood collection device (approved by Health Canada), or by venipuncture.^45^ For children, an anaesthetic cream, Ametop GEL 4% (tetracaine hydrochloride gel), will be offered to be applied to the puncture site 30 min before blood collection.^46^

In patients consenting to participate in the DIVE or DIVE2 sub-study, supplementary biological samples will be collected during the methacholine visit, including the additional blood samples, nasosorption test for NELF and urine. The total volume of blood drawn from a child will adhere to Quebec’s standards for maximum blood volumes based on weight.^36^

Participants will then undergo spirometry or BPT with methacholine, depending on the visit, as per the diagnostic procedure.

Spirometry will be performed according to American Thoracic Society (ATS)/European Respiratory Society (ERS) standards, with reference values derived from the Global Lung Initiative 2022 race-neutral prediction equations.^47^ Patients will have been informed in advance of the need to withhold treatment as outlined in table 1.^48 49^

**Table 1:** Recommended inhaler washout period prior to testing^48 49^

	Washout period	
	Spirometry	Bronchial provocation test
Short-acting β₂-agonists	> 4 hours	> 6 hours
Short-acting muscarinic antagonists	> 12 hours	> 12 hours
Long-acting β₂-agonists	> 36 hours	> 48 hours
Long-acting muscarinic antagonists	> 48 hours	> 1 week

Diagnostic criteria for asthma include:

1. Bronchodilator Reversibility Test: An increase in FEV₁ of ≥12% from baseline for all participants, with an additional criterion of ≥200 mL absolute improvement required for adults (≥18 years). Bronchodilator administration will be standardised using salbutamol 400 mcg delivered via spacer device.
2. Methacholine BPT: Airway hyper-responsiveness, defined as a provocative dose causing a 20% decrease in FEV₁ (PD₂₀) of <400 mcg (equivalent to a provocative concentration (PC₂₀) of<16 mg/mL). While we acknowledge the more stringent European threshold (PD₂₀ ≤200 mcg, equivalent to PC₂₀ ≤8 mg/mL), we will adhere to our institutional protocols.^1^,^3^ The latter (European) diagnostic threshold will be evaluated through pre-planned sensitivity analyses.

At the end of the visit, all participants will complete a satisfaction questionnaire regarding the inflammatory biomarker testing and overall visit experience.

Participants will be followed longitudinally via email correspondence at 4, 8 and 12 months following diagnostic confirmation. At each time point, participants will complete questionnaires covering the period since the last follow-up. Data will be collected on prescribed and dispensed medications, medication use, symptom evolution, exacerbations, health-related quality of life (measured by the EQ-5D-5L), frequency of exacerbations, healthcare utilisation and asthma control (assessed using the ACQ-5 and ACT).

All collected variables are indicated in online supplemental table S2.

### Intervention

The interpretation of respiratory function tests and biomarkers will be performed by the principal investigator pulmonologist at each centre or by trained designated collaborating pulmonologists by following these models:

1. High-risk inflammatory asthma is defined by FeNO ≥25 ppb or BEC ≥300 cells/µL in participants ≥12 years (≥20 ppb or ≥300 cells/µL in children aged 6 to <12 years)^15 50^ : one or more elevated biomarkers will be interpreted as a high risk of asthma exacerbation. If spirometry is non-diagnostic, BPT will be prioritised (<2–4 weeks).
2. Low risk inflammatory asthma is defined by FeNO <25 ppb and BEC <300 cells/µL (<20 ppb or <300 cells/µL in children aged 6–<12 years)^15 50^: this will be interpreted as a low risk of asthma exacerbation. If spirometry is non-diagnostic, BPT will be scheduled according to the standard waiting list.

Results of biomarkers will be integrated on the medical report indicating the probability (high/low) of asthma exacerbation and suggested management with either ICS taken as-needed (T2-low) or regularly (T2-High) ICS if clinically appropriate, which will be forwarded to the referring physician.

**Figure 2:**
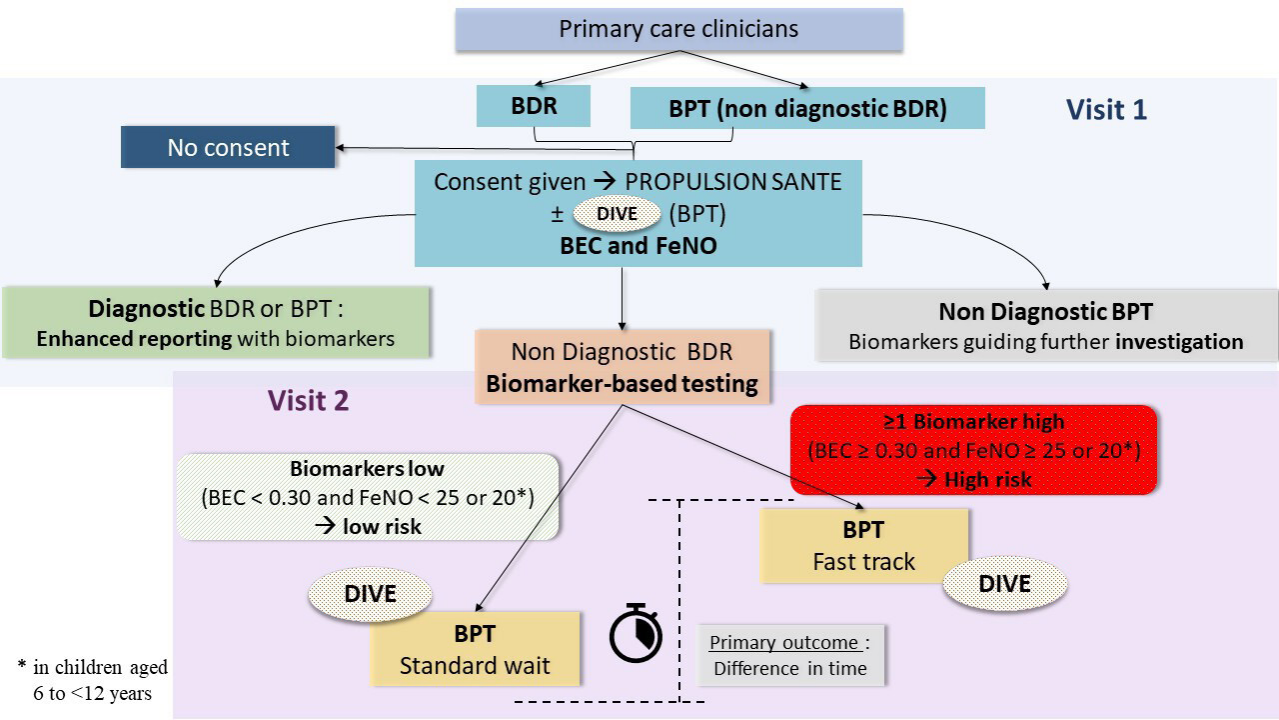
Flow Diagram. Patients with suspected asthma are referred by primary care for pulmonary function testing (BDR or BPT). Those who consent to participate in the study will undergo BEC and FeNO testing. Patients with normal spirometry results and at least one elevated inflammatory marker will have their methacholine challenge test prioritised. If no inflammatory markers are elevated, the BPT will be scheduled within standard timelines. Patients undergoing a BPT—either directly on referral or after an initial BDR—will be invited to participate in the translational research studies, DIVE for adults or DIVE2 for children. Patients with a confirmed diagnosis of asthma (based on spirometry or BPT) will also be stratified according to their inflammometry results, which will be included in the report sent to the referring physician. BEC, blood eosinophil count; BDR, bronchodilator reversibility; BPT, bronchial provocation testing; FeNO, fraction exhaled of nitric oxide.

The patient test pathway based on their respiratory test and the results of the inflammometry assessment is summarised in figure 2.

### Data and sample management

Participant data will be collected prospectively in REDCap and stored on encrypted servers with restricted access hosted at the University of Sherbrooke.^33^ Follow-up will be conducted via REDCap emails with reminders after 15 days of no response.

BEC will be performed in each institution’s clinical laboratory (complete blood count with differential), after sampling in an EDTA tube. Blood samples will be processed within 4 hours of collection according to standardised protocols. For participants enrolled in the complementary DIVE or DIVE 2 studies, additional sample processing will include plasma separation, peripheral blood mononuclear cell separation and preservation of whole blood and DNA for future methylation analyses, following standardised biobanking protocols: inflammatory protein quantification will be performed on serum and NELF for IL-4, IL-5, IL-13, IL-33, thymus associated regulatory cytokine (TARC), thymic stromal lymphopoietin (TSLP), eotaxin-3, interferon gamma (IFNγ) and tumour necrosis factor (TNF) using multiplex electrochemiluminescent assays (MesoScale Discovery) and ELISA.

### Outcomes measures

#### Primary Outcome

The primary outcome, diagnostic delay, will be defined as the interval between primary care requisition for pulmonary function testing and the last confirmatory diagnostic test conducted (BDR or BPT). This continuous outcome will be analysed longitudinally and stratified by biomarker-guided diagnostic strategies, also adjusting for the time of inclusion in the study. Sensitivity analyses will also examine diagnostic delay for FeNO and blood eosinophils considered separately.

#### Secondary Outcomes

1. Diagnostic accuracy of inflammometry assessed through receiver operating characteristic (ROC) curve analyses using FeNO levels, BEC and their combined values, with BPT as gold standard. Clinically relevant thresholds will be identified to optimise rule-in capacity for asthma diagnosis in primary care, targeting ≥90% of specificity and a positive likelihood ratio (LR+) ≥10 in children, adolescents and adults (as three distinct subgroups).
2. Assessment of inflammatory status on asthma control, evaluated pre-diagnosis and post-diagnosis using the ACQ-5, ACT and EQ-5D-5L questionnaire scores. Additional measures include the number of exacerbations, prescribed and dispensed medications and the frequency of specialised asthma consultations collected from clinical records, drug claims and patient electronic reports at baseline and at 4-month, 8-month and 12-month follow-up.
3. Assessment of health benefits in terms of QALY, budget impact and economic efficiency (evaluated using incremental cost-effectiveness ratio (ICER) and Incremental cost-utility ratio (ICUR)) of integrating inflammatory biomarkers into primary care asthma diagnosis.
4. The evaluation of annualised carbon dioxide (CO2) emissions from inhalers dispensed to non-asthmatic individuals awaiting their diagnostic tests was done based on drug claims and Cascades Canada environmental data.^27^ Environmental sustainability assessment of biomarker-enabled pathways will be explored.
5. Descriptive analysis of patient satisfaction regarding inflammometry and the overall experience of diagnostic procedures, with responses stratified by age (6–<12, 12–<18, ≥18) and diagnostic test (BDR or BPT) and final asthma diagnosis status.

### Translational outcomes

Evaluation of the association between inflammatory biomarkers and underlying inflammatory mediators through participation in the DIVE and DIVE2 translational sub-studies. Findings will be linked to the final asthma diagnosis. The remaining biobanked samples will serve future translational analyses for biomarker discovery in asthma diagnosis, for example, based on epigenetic or metabolomic signatures.

### Statistical methods

Data will be independently analysed by a biostatistician from the CHUS using R software (V. 4.2.0 or later, R Foundation for Statistical Computing, Vienna, Austria) in collaboration with a health economist. Missing data will be documented, and multiple imputation by chained equations will be considered for data missing at random when <20% of data is missing. Statistical significance will be determined using a two-sided alpha level of 0.05. Adjustments for multiple comparisons with the Benjamini-Hochberg procedure will be applied where necessary, including for subgroup analyses on the primary outcome.^51^

All primary analyses will also be stratified by pre-specified subgroups: study site, age categories (6–<12 years, 12–<18 years, ≥18 years), biological sex, ethnicity, smoking status and other potential confounding variables related to inflammatory biomarker measurement. Additionally, we will perform sensitivity analyses excluding active smokers. Categorical variables will be presented as counts with corresponding percentages, while continuous variables will be reported as either means with standard deviations (for normally distributed data) or medians with interquartile ranges (for non-normally distributed data). For comparative analyses between groups, we will employ independent t-tests or Mann-Whitney U-tests for continuous variables based on distribution, and χ^2^ tests or Fisher’s exact tests for categorical variables, as appropriate based on expected cell frequencies.

For the primary outcome of waiting time differences, the baseline diagnostic delay, prior to the project implementation, will be established based on the time elapsed between the initial request for an asthma diagnostic test made by primary care and the performance of that test. During the intervention, diagnostic delays will be monitored prospectively, both overall and stratified by inflammatory status. We will apply linear mixed-effects models with restricted maximum likelihood estimation. These models will incorporate (high-risk vs low-risk), study period at time of inclusion, and the number of awaiting tests as fixed effects. Random effects will account for within-centre clustering and repeated measurements. In addition, Kaplan-Meier survival curves will be constructed to visualise the probability of remaining undiagnosed over time for both high-risk and low-risk inflammatory phenotypes. These curves will allow for a graphical representation of differences in diagnostic efficiency between groups. Cox proportional hazards regression will be used to identify significant predictors of waiting time with HRs and 95% CIs reported.

Diagnostic performance in the secondary outcome will be evaluated through ROC analysis, calculating the area under the curve (AUC) with 95% CIs for each individual marker and for their combined use. Sensitivity, specificity, positive and negative predictive values and likelihood ratios will be calculated with their respective 95% CIs.

Health utility values will be derived from EQ-5D questionnaires^39 52 53^ completed at baseline and follow-up visits. Quality-adjusted life years will be calculated using the AUC method, connecting utility values across measurement time points. This approach integrates health-related quality of life over the 12-month follow-up period. To assess the financial implications of implementing FeNO testing within the provincial healthcare framework, we will develop a budget impact model for the MSSS. Environmental and satisfaction-related outcomes will be analysed using descriptive statistics. CO₂ emissions from inhalers dispensed to non-asthmatic individuals will be estimated based on Cascades Canada data,^27^ and patient satisfaction with inflammometry will be assessed using structured questionnaires.

Additional exploratory clinical outcomes will be analysed using appropriate descriptive statistics in accordance with distribution. Linear and logistic regression analyses will associate the diagnostic outcome, T2 biomarkers and blood/NELF inflammatory mediators.

Sensitivity analyses will be conducted with the exclusion of paediatric-only study sites (CHUSJ and MCH-MUHC) and with different diagnostic asthma criteria (eg, BDR with 10% in % predicted FEV1 or FVC values, BPT with PD20 <200 µg).

### Sample size

Approximately 3000 diagnostic tests for suspected asthma are currently pending across the three participating centres (CHUS, CHUSJ and MCH), including around 1500 paediatric tests. Propulsion Santé was funded for 12 months of full-time recruitment (convenience sampling). Based on this pre-existing waitlist and expected referral rates, the Propulsion Santé study aims to recruit 1500 participants aged 6 years and older in the three centres. This sample size will allow us to detect a minimal effect size of 0.07 (Cohen’s f), assuming a significant level of 5%, a power of 80% and a correlation of 0.5 among repeated measures. The calculation was performed using a power analysis for a repeated measures ANOVA with a between-subjects factor in G*Power v 3.1.9.4. Of course, this sample size exceeds our minimum requirement, providing greater assurance in detecting the expected effects, but will also provide a unique opportunity to evaluate diagnostic delays and outcomes in real-world conditions. In addition, the findings will contribute to an ongoing meta-analysis on the diagnostic performance of inflammatory biomarkers.^54^

For the DIVE and DIVE 2 translational substudies (designed prior to PROPULSION SANTÉ), a sample size of 123 participants was originally calculated to provide 90% power to assess the diagnostic performance of three ROC curves. The sample size assumes a 2:1 ratio of asthma-positive to asthma-negative participants, allowing the analysis of three ROC curves, each targeting area under the curve (AUC) values ≥ 0.7, which are significantly different from the null hypothesis (AUC = 0.5), using a two-sided alpha level < 0.016, adjusted for multiple comparisons across three biomarkers.

### Ethics and dissemination

All study protocols have been reviewed and approved by a central Research Ethics Board (#MP-31-2025-5593; MP-31-2024-5346; 2023–4791). The results will be disseminated through presentation at an international conference and publication in a peer-reviewed journal, reported according to the STROBE^55^ and STARD Statements,^56^ and including disaggregation of results according to biological sex and gender.

### Discussion

This study addresses a critical gap in the literature by prospectively evaluating the implementation of inflammatory biomarkers in a real-world primary care referral pathway across both adult and paediatric populations. Beyond its scientific contribution, our protocol will optimise clinical resources by reducing the backlog of approximately 3000 respiratory function tests, currently awaiting completion across the three participating centres, through the engagement of study-dedicated respiratory therapists.

We anticipate that this study will demonstrate the utility of inflammatory biomarkers as effective triage and prioritise instruments for BPT. Given the high rate of false positives in respiratory testing as well as the substantial rate of clinical misclassification in Canada (between 33% and 45% in adults and children),^20 22^ our study will calculate optimal biomarker thresholds to achieve 90% specificity, positive predictive value (PPV) exceeding 80% and LR+ greater than 10—parameters essential for reliable diagnostic confirmation.^57^ In effect, this strategy sacrifices sensitivity in favour of specificity, leading to high PPV and LR+.

Existing literature supports the relevance of our approach. Li *et al* conducted a large retrospective cohort study involving 7463 adult patients with suspected asthma in secondary care. Among the 2349 patients with complete biomarker data, concurrent elevation of FeNO (> 40 ppb) and BEC (>0.3×10ˆ9 cells/L) was associated with a diagnostic specificity greater than 95%.^58^ Similarly, Nekoee *et al* demonstrated that combining biomarkers with spirometry improved diagnostic accuracy (AUC 0.82, 95% CI 0.78 to 0.86),^59^ reinforcing the relevance of inflammometry in routine assessment. Additionally, the predictive value of both BEC and FeNO has been demonstrated in adults,^812 15 16 19 60^,^70^ and to a lesser extent in the paediatric^50^ populations, with significant clinical implications for early intervention and treatment adjustment. Furthermore, these biomarkers are valuable tools for therapeutic decision-making, as elevated values are associated with a better response to anti-inflammatory therapies.^71^,^75^ As modifiable indicators, the inflammatory signals provided by BEC and FeNO highlight the importance of early measurement to better prioritise patients requiring timely inflammation control.^15 61 62 64^

We acknowledge potential methodological limitations. The multicentre design necessitates rigorous harmonisation of practices across sites. The risk of missing data—especially in paediatric patients due to refusal of blood sampling or difficulty in FeNO measurement among children aged 6–8 years—is a concern; this will be minimised by offering a relatively painless capillary blood sampling (which has shown a failure rate below 5%)^76^ or by applying topical analgesia for venipuncture. For FeNO, success rates increase from approximately 40% at age 5 to nearly 100% by age 10.^42 77^ Additionally, the use of 6-s FeNO measurements is expected to further improve feasibility in children aged 6–8 years. Sensitivity analyses using FeNO or blood eosinophils alone will reflect what can be achieved in real-world conditions if one of the biomarkers is not available. Additional limitations include the intrinsic variability of biomarkers related to recent infections or medication use, management of comorbidities that may influence biomarkers and the complexity of economic evaluation across different centres. Nonetheless, this diversity enhances the generalisability of findings across Quebec and potentially other Canadian provinces. Our findings must be contextualised within the evolving landscape of precision medicine in respiratory care. Pavord *et al* have emphasised the clinical and economic benefits of biomarker-guided treatment algorithms in severe asthma, demonstrating reduced exacerbations and improved quality of life through targeted biological therapies.^61^ Similarly, the CAPTAIN study highlighted how biomarker stratification can predict treatment response, potentially transforming clinical decision-making from earlier disease stages.^71^ In this way, our approach brings the principles of precision medicine into the diagnostic process by focusing on identifiable and modifiable traits in an understudied population, that is, individuals with predominantly non-severe asthma issued from the primary/community care. It helps reduce uncertainty in asthma diagnosis by moving from a standard approach to a more personalised assessment using early measurable biological markers.^78^

Our study evaluates the effectiveness of current asthma diagnostic methods in Canada and proposes improvements in Canada. The anticipated outcomes are multifaceted: (1) improved equity in healthcare access through better prioritisation; (2) enhanced patients outcomes by enabling early identification and treatment of individuals with elevated inflammation, thereby reducing exacerbations and lung function decline; (3) organisational and economic efficiency through reduction of costly tests and unnecessary consultations; (4) generation of novel paediatric data; (5) integration of environmental considerations through reduction of inhaler-related emissions.^28 79^

An additional strength of the present project is the integration of two complementary translational substudies—DIVE (in adults) and DIVE 2 (in children)—which provide a unique opportunity to link clinical inflammatory phenotypes with underlying molecular profiles through the collection of NELF and serum samples, and the quantification of key inflammatory proteins (eg, alarmins, interleukins and chemokines). Patients are not pre-selected by T2 phenotype, as these studies are designed as real-world primary care studies. Maintaining the distribution of T2-high and T2-low patients, as observed in the general population, allows assessment of biomarker performance under real-world conditions. These sub-studies will help identify potential biomarkers associated with type 2 inflammation and final asthma diagnosis. By incorporating both paediatric and adult populations, this integrated approach enhances the relevance and applicability of findings and supports the development of precision diagnostic tools that are biologically grounded and clinically actionable.

## Conclusion

This innovative multicentre observational study is the first to assess the real-world application of inflammatory biomarkers in the asthma diagnostic test pathway among both paediatric and adult populations referred from primary care. By prospectively evaluating FeNO and BEC and comparing with conventional lung function tests, we aim to inform a more efficient and evidence-based system for prioritising suspected asthma cases.

Biomarker-guided stratification appears promising to reduce diagnostic delays in patients with inflammatory phenotypes, thus minimising unnecessary treatment in non-asthmatic individuals and improving clinical outcomes through recommendations for targeted interventions. The study will provide solid evidence to refine age-specific biomarker cut-offs for diagnostic purposes, addressing the current scarcity of validated thresholds in the paediatric population and improving understanding of biomarker performance across diverse demographic groups.

By enhancing diagnostic accuracy and resource efficiency while supporting personalised care, this project will advance the principles of precision medicine. It integrates accessible biomarker tools into routine clinical practice, offering a scalable strategy to streamline diagnosis, control healthcare costs, potentially reduce the environmental impact of unnecessary inhalers used during diagnostic delays and foster a more sustainable and cost-effective approach to respiratory care.

## Supplementary material

10.1136/bmjresp-2025-003750 online supplemental file 1

## Data Availability

Data are available upon reasonable request.
