# Supplementary material for: Integrating blood eosinophils and exhaled nitric oxide (FeNO) in asthma diagnostic pathways for adults and children: the PROPULSION SANTÉ observational study with translational sub-studies (DIVE, DIVE2)—protocols
Source: BMJ Open Respir Res. 2025 Nov 27;12(1):e003750. doi: 10.1136/bmjresp-2025-003750 (PMC12666203; doi:10.1136/bmjresp-2025-003750)
Supplement: online supplemental file 1 [file bmjresp-12-1-s001.docx]

# Supplementary materials

# Table S1

**Research Protocol Variables Table: Asthma Biomarkers Study**

| **Variable** | **Description** | | **Data Source** | | **Scale** | | **Analysis Method** | |
| --- | --- | --- | --- | --- | --- | --- | --- | --- |
| **Subject characteristics** | |  | |  | |  | |  |
| Age | Patient age at time of assessment | | Respiratory physiology request | | Years | | Descriptive: mean or standard deviation | |
| Sex | Male or Female | | Respiratory physiology request | | Binary | | Descriptive | |
| Ethnicity | Caucasian; African American; Latino American; Asian; Native American; Other | | Respiratory physiology request | | Categorical | | Descriptive | |
| Height | Patient height | | Respiratory physiology request | | cm | | Descriptive | |
| Weight | Patient weight | | Respiratory physiology request | | kg | | Descriptive | |
| BMI | Body Mass Index | | Calculated | | kg/m² | | Descriptive | |
| **Medical history** | |  | |  | |  | |  |
| Relevant medical history | COPD; unstable heart disease; cystic fibrosis; bronchiectasis; recent respiratory infection (last 6 weeks) | | Respiratory therapist questionnaire | | Nominal list | | Descriptive | |
| Allergy history | Eczema, allergic rhino-conjunctivitis, food allergy, and past allergy tests | | Respiratory therapist questionnaire | | Nominal list | | Descriptive | |
| **Biomarkers** | |  | |  | |  | |  |
| BEC | Quantification of blood eosinophils | | Standard blood sample and/or Tasso | | Absolute values (cells ×10⁹/L) | | ROC curve with AUC calculation | |
| FeNO | Fractional exhaled Nitric Oxide | | Niox Vero – Circassia, Oxford/UK | | Continuous values (ppb) | | ROC curve with AUC calculation | |
| **Lung function** |  | |  | |  | |  | |
| PD20 (Gold standard) | Provocative dose causing 20% fall in FEV₁ | | Methacholine challenge test | | mcg | | Reference standard | |
| FEV₁ | Forced Expiratory Volume in 1 second | | Pre and post bronchodilator spirometry in medical record; if unavailable, pre-challenge test spirometry | | Liters and % predicted | | Descriptive | |
| FVC | Forced Vital Capacity | |  |  | Liters and % predicted and Z-score | | Descriptive | |
| FEV₁/FVC | Ratio of FEV₁ to FVC | |  |  | Observed ratio and Z-score | | Descriptive | |
| Environmental exposures | |  | |  | |  | |  |
| Smoking status | Active smoker, ex-smoker, non-smoker, passive smoking, pack-years | | Respiratory therapist questionnaire | | Categorical and continuous value in pack-years | | Descriptive | |
| Other inhaled substances | E-cigarette, inhaled cannabis, other inhaled drugs | | Respiratory therapist questionnaire | | Categorical and semi-quantitative (frequency of use) | | Descriptive | |
| Clinical outcomes | |  | |  | |  | |  |
| ACQ, ACQ-5 Score | Assessment of asthma control | | Questionnaire | | Continuous value (<1.5: good control) | | Descriptive | |
| ACT Score | Assessment of asthma control | | Questionnaire | | Continuous value (>19: good control) | | Descriptive | |
| Utility (Eq-5d Score) | Health-related quality of life | | Questionnaire (Eq-5d/Eq-5d-Y) | | Categorical and continuous (0-100) | | Descriptive | |
| **Healthcare utilization & efficiency metrics** | | | |  | |  | |  |
| Cost | Costs associated with diagnostic algorithms | | Multiple sources (MSSS reimbursement tables, consumables, staffing, interpretation) | | Continuous value ($) | | Descriptive | |
| Diagnostic delay | Time from referral to diagnosis | | Medical records | | Days | | Descriptive | |
| Methacholine test time | Duration of challenge test | | Test records | | Minutes | | Descriptive | |
| **Stratification metrics** | |  | |  | |  | |  |
| Risk categorization | Patient distribution in 3×3 grid by FeNO and eosinophil thresholds | | Calculated from biomarker values | | % of patients per category | | Descriptive | |
| **Environmental impact** | |  | |  | |  | |  |
| Inhaler carbon footprint | Carbon emissions from inhalers during diagnostic period | | Prescription records and CASCADES Canada values | | kg CO₂e | | Descriptive | |
| Healthcare carbon footprint | Carbon emissions from healthcare visits | | Visit records and literature values | | kg CO₂e | | Descriptive | |
| **User experience** | |  | |  | |  | |  |
| Patient appreciation | Satisfaction with inflammometry measures | | Likert questionnaire (patients/guardians) | | Categorical | | Descriptive | |
| Longitudinal outcomes | | |  | |  | |  | |
| Asthma follow-up | Medication use, symptoms, quality of life, exacerbations | | Questionnaires (Eq-5D-Y, ACT, ACQ) at 4, 8, and 12 months; reMEd database | | Categorical and continuous | | Descriptive | |
| Healthcare consumption | Medical visits for asthma and related diagnoses | | RAMQ and MEDECHO | | Categorical and continuous | | Descriptive | |

ACQ, Asthma Control Questionnaire; ACT, Asthma Control Test; AUC, Area Under the Curve, COPD, Chronic Obstructive Pulmonary Disease; Eq-5D, EuroQol 5 Dimensions; FEV1, Forced Expiratory Volume in 1 Second; FVC, Forced Vital Capacity; kgCO₂, Kilograms of Carbon Dioxide; mcg, Microgram, MSSS, Ministère de la Santé et des Services sociaux *(Ministry of Health and Social Services*), PD20, Provocative Dose Causing a 20% Fall in FEV1; ppb, Parts Per Billion; RAMQ, Régie de l'assurance maladie du Québec, ROC, Receiver Operating Characteristic
